# High BMI is significantly associated with positive progesterone receptor status and clinico-pathological markers for non-aggressive disease in endometrial cancer

**DOI:** 10.1038/bjc.2011.46

**Published:** 2011-02-22

**Authors:** K K Mauland, J Trovik, E Wik, M B Raeder, T S Njølstad, I M Stefansson, A M Øyan, K H Kalland, T Bjørge, L A Akslen, H B Salvesen

**Affiliations:** 1 Department of Obstetrics and Gynaecology, Haukeland University Hospital, 5021 Bergen, Norway; 2 Department of Clinical Medicine, University of Bergen, Bergen, Norway; 3 Section for Pathology, The Gade Institute, University of Bergen, Bergen, Norway; 4 Department of Pathology, Haukeland University Hospital, Bergen, Norway; 5 Department of Microbiology, Haukeland University Hospital, Bergen, Norway; 6 Department of Public Health and Primary Health Care, University of Bergen, Bergen, Norway; 7 Norwegian Institute of Public Health, Bergen, Norway

**Keywords:** body mass index, endometrial carcinoma, prognosis, progesterone receptor

## Abstract

**Background:**

Endometrial cancer incidence is increasing in industrialised countries. High body mass index (BMI, kg m^−2^) is associated with higher risk for disease. We wanted to investigate if BMI is related to clinico-pathological characteristics, hormone receptor status in primary tumour, and disease outcome in endometrial cancer.

**Patients and methods:**

In total, 1129 women primarily treated for endometrial carcinoma at Haukeland University Hospital during 1981–2009 were studied. Body mass index was available for 949 patients and related to comprehensive clinical and histopathological data, hormone receptor status in tumour, treatment, and follow-up.

**Results:**

High BMI was significantly associated with low International Federation of Gynaecology and Obstetrics (FIGO) stage, endometrioid histology, low/intermediate grade, and high level of progesterone receptor (PR) mRNA by qPCR (*n*=150; *P*=0.02) and protein expression by immunohistochemistry (*n*=433; *P*=0.003). In contrast, oestrogen receptor (ER*α*) status was not associated with BMI. Overweight/obese women had significantly better disease-specific survival (DSS) than normal/underweight women in univariate analysis (*P*=0.035). In multivariate analysis of DSS adjusting for age, FIGO stage, histological subtype, and grade, BMI showed no independent prognostic impact.

**Conclusion:**

High BMI was significantly associated with markers of non-aggressive disease and positive PR status in a large population-based study of endometrial carcinoma. Women with high BMI had significantly better prognosis in univariate analysis of DSS, an effect that disappeared in multivariate analysis adjusting for established prognostic markers. The role of PR in endometrial carcinogenesis needs to be further studied.

Endometrial cancer is the most common gynaecological malignancy in industrialised countries (Parkin *et al*, 2005), and the incidence has been increasing over the last decades (Cancer Registry of Norway, 2009). Obesity is a known risk factor for disease development with a higher risk with increasing body mass index (BMI, kg m^−2^) (Schouten *et al*, 2004; Bjorge *et al*, 2007). It has recently been shown that morbidly obese women (BMI⩾40) have a six-fold increase in risk of disease development (Lindemann *et al*, 2008). This is presumably related to unopposed oestrogen exposure. After menopause, the ovaries and adrenal glands continue to produce androstenedione, which is converted to oestrone in adipose tissue by the aromatase enzyme. This weaker oestrogen may stimulate chronic endometrial proliferation and cancer development after menopause (Kaaks *et al*, 2002). Tumours arising in such hyper-oestrogenic environment are typically type I endometrial carcinomas, characterized by endometrioid histology, low grade, hormone receptor-positive status, and good prognosis. In contrast, tumours of type II are typically not oestrogen driven, of non-endometrioid histology, high grade, with loss of hormone receptors and poor prognosis (Bokhman, 1983; Amant *et al*, 2005). However, the prognostic value of the distinction between type I and type II endometrial cancer is limited, as up to 20% of type I endometrial cancers recur and 50% of type II cancers do not (Engelsen *et al*, 2009). Diagnostic accuracy and reproducibility of histological subtyping is a challenge. Therefore, there is need for new prognostic markers. Even though it is well established that obesity gives higher risk for endometrial cancer, studies relating BMI to clinical and histopathological markers and survival are scarce, and partly contradictive (Anderson *et al*, 1996; Duska *et al*, 2001; von Gruenigen *et al*, 2006; Temkin *et al*, 2007; Munstedt *et al*, 2008; Jeong *et al*, 2010). In particular, no previous studies have identified molecular markers for hormone receptor status in the tumour tissue related to BMI.

On this background, we have investigated the relationship between BMI and a large panel of clinical and histopathological data, hormone receptor status in primary tumours, and disease outcome in a large population-based endometrial carcinoma series.

## Patients And Methods

### Patient series

The patient series include 1129 women primarily treated for endometrial carcinoma at Haukeland University Hospital during the period 1981 through 2009. This is the referral hospital for Hordaland county, with ∼475 000 inhabitants, representing about 10% of the Norwegian population (SSB, 2010). The endometrial cancer incidence rate and prognosis in this area are similar to data for the total population (Cancer Registry of Norway, 2009).

Information concerning height, weight, age, menopausal status, International Federation of Gynaecology and Obstetrics (FIGO) stage, histological subtype and grade, treatment, and follow-up was collected by review of the medical records and through correspondence with the primary physicians. In all, 91% of the women underwent hysterectomy with bilateral salpingo-oophorectomy as primary treatment and were classified according to the FIGO 1988 criteria (Mikuta, 1993). If surgical treatment was contraindicated, the staging was based on the available information from curettage results, clinical examination, chest X-ray, and abdomino-pelvic CT.

Follow-up time was defined as the time interval between date of primary diagnosis and date of death or last follow-up. The median follow-up time was 4.9 years (range 0.01–23.2). In all, 223 patients (20%) died from endometrial carcinoma during the follow-up period, while 207 (18%) died from other causes. These data were cross-checked with information from the Cancer Registry of Norway and the Register of Statistics Norway. Last follow-up was 20 December 2009.

Body mass index was calculated as weight (kg) divided by squared height (m^2^), both measured at the time of diagnosis. These data were available for 949 patients (84%). For the statistical analyses on BMI we used the quartiles for the data set as cut points, as well as the established WHO classification system; BMI under 18.5 (underweight), between 18.5 and 24.9 (normal), between 25 and 29.9 (overweight), and >30 (obese). Height and weight of outliners (BMI<15 and BMI>50, *n*=7) was double-checked. All analyses were also performed excluding these; this did not affect any of the conclusions.

### Immunohistochemistry

Formalin-fixed paraffin-embedded tumour specimens were mounted in tissue microarrays (TMAs) as previously described (Hoos *et al*, 2001; Stefansson *et al*, 2004). Briefly, TMA was constructed by identifying the area of highest tumour grade on HE-stained slides, followed by punching out three tissue cylinders from the selected areas of the donor block and mounting these into a recipient paraffin block using a custom-made precision instrument (Beecher Instruments, Silver Spring, MD, USA). Immunohistochemical staining for receptor status was assessed for oestrogen- and progesterone receptors (ER*α* and PR) and available for 437 and 433 patients for ER*α* and PR, respectively (38% of study population). The method for immunohistochemical staining was as previously described, using the lower quartile to define receptor loss (Engelsen *et al*, 2008b).

### qPCR analysis

From a subset of 150 patients (13%), fresh frozen tumour tissue was collected prospectively and was available for mRNA analysis in parallel with the immunohistochemical staining. Total RNA was extracted using the RNeasy kit (Qiagen, Hilden, Germany), with quality control and method for data processing as previously reported (Engelsen *et al*, 2008a; Salvesen *et al*, 2009). mRNA expression levels in tumours for ER*α* and PR were investigated by qPCR using the TaqMan Low Density Array technique (Engelsen *et al*, 2008a).

### Statistical methods

Body mass index in WHO categories was applied to assess the distribution of various clinico-pathological variables, using the Pearson's *χ*^2^-test. Hormone receptor status in primary tumour in relation to BMI was assessed by the Mann–Whitney *U*-test. Univariate survival analyses for disease-specific survival (DSS) and overall survival (OS) were performed using the Kaplan–Meier method (log-rank test). The Cox proportional hazard regression analysis was applied to evaluate the prognostic impact of BMI adjusted for the established prognostic markers in endometrial carcinoma. We compared the distribution of clinico-pathological variables and prognosis for patients with available data for BMI to patients where these data were missing (16%). Women lacking BMI data were older, with median age 69.3 years compared with 65.2 years for the group where BMI was registered, *P*=0.004 (Mann–Whitney *U*-test). No other significant differences were identified. The statistical software PASWStatistics18.0 was used for data analyses (SPSS Inc., Chicago, IL, USA).

The study was approved by the IRB (NSD 15501, REK III nr 052.01).

## Results

### High BMI associates with clinico-pathological markers for non-aggressive disease

**Figure 1:**
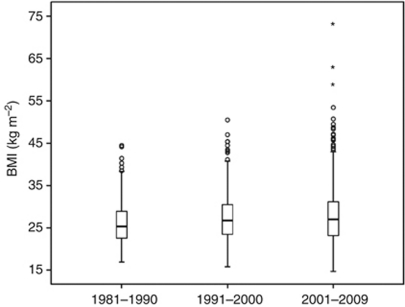
Distribution of BMI for endometrial carcinoma patients treated in one defined region in Norway (Hordaland county) in the periods 1981–1990, 1991–2000, and 2001–2009. Median BMI and range increase significantly from 25.3 (16.9–44.5) to 26.7 (15.8–50.5) and 26.9 (14.7–73.0) for the time periods studied, *P*=0.002 (Kruskal–Wallis test). 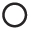
=minor outliers and 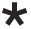
=major outliers.

The median BMI at diagnosis was 26.4 (range 14.7–73.0), with significantly increasing BMI throughout the study period, *P*=0.002 (Figure 1). There was a significant association between BMI and patient age at diagnosis, FIGO stage, and histological subtype, as shown in Table 1. The proportion of patients with BMI<25 was larger in the lower and upper age quartiles compared with BMI⩾25, whereas there was a tendency for the patients of the middle age quartiles to be overweight or obese. The proportion of normal/lean patients was larger for FIGO stages III and IV compared with FIGO stages I and II. High BMI was also associated with endometrioid histology. There was no significant association between BMI and menopausal status nor BMI and grade. Also, there was no significant difference in number of performed lymphadenectomies related to BMI (*P*=0.99), but a tendency to more adjuvant therapy given to patients with BMI<25 (*P*=0.06).

### High BMI associates with positive PR status in tumour

When investigating biomarkers for receptor status in tumours related to BMI we found that patients with PR-negative tumours (by IHC) had lower median BMI compared with the patients who had PR-positive tumours, median 25.5 *vs* 26.9, respectively (*P*=0.003, Mann–Whitney *U*-test). We did not find any significant correlation between BMI and ER*α* status in tumours (*P*=0.08) (Table 1). To further validate this finding, we examined a subset of 150 fresh frozen patient samples for mRNA expression levels for hormone receptors by qPCR. This confirmed a significantly higher mRNA expression level for PR in patients with BMI>25 compared with patients with lower BMI (*P*=0.02, Mann–Whitney *U*-test). For ER*α*, no such association with BMI was observed for mRNA expression levels (*P*=0.21). Loss of ER*α* and PR (by IHC) was associated with postmenopausal status (*P*=0.01 and *P*=0.006, respectively, Pearson's *χ*^2^-test).

### BMI and prognosis

#### Univariate analysis

**Figure 2:**
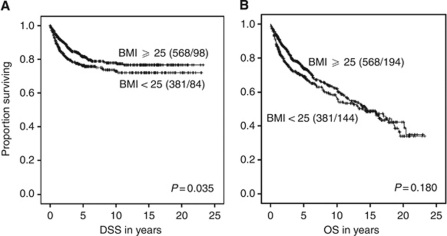
Univariate survival plot by Kaplan–Meier for estimation of DSS (**A**) and OS (**B**) in patients with endometrial carcinoma related to BMI. The total number of patients in each group is followed by number of deaths, given in parentheses; *P*-value based on the Mantel–Cox test.

The established clinico-pathological variables showed, as expected, a highly significant impact on DSS, as listed in Table 2. There was a trend towards better prognosis for patients with higher BMI in univariate analysis (Table 2). Patients being overweight/obese *vs* normal/underweight as defined by the WHO had better DSS, with a 5-year survival of 82% for women with BMI⩾25 compared with 76% for BMI<25 (*P*=0.035; Figure 2A; Table 2). For OS, we found that patients with BMI<25 had a 5-year survival of 69% compared with 74% for patients with BMI⩾25 (*P*=0.18; Figure 2B). In the OS analysis, we also see a pattern of diminishing survival difference between the two BMI groups >10 years after diagnosis. This may relate to the higher risk of developing other diseases for overweight women, being more important than the risk for cancer-related deaths >10 years after diagnosis.

#### Multivariate analysis

The survival effect of BMI observed in univariate analysis for DSS disappeared when adjustment was made for age at diagnosis (continuous variable), FIGO stage, histological subtype, and grade in the Cox multivariate regression analysis as listed in Table 3. Adjusted HR for BMI<25 *vs* ⩾25 was 0.93 (CI 0.68–1.27, *P*=0.65). When BMI was applied as a continuous variable in the same Cox model, we found a similar insignificant HR for BMI of 1.01 (CI 0.98–1.04), and pattern for the other variables with independent impact for FIGO stage and age only. In contrast, when using OS as end point in the Cox model, we found that BMI had independent impact on prognosis when introduced as a continuous variable with an HR 1.02 (CI 1.00–1.04, *P*=0.035). FIGO stage and age were also independent predictors of prognosis (*P*<0.0001 for both), while histology was of borderline significance (*P*=0.053) and grade was non-significant (*P*=0.166).

## Discussion

To our knowledge, this is the most comprehensive study of clinico-pathological variables to date. It is also the largest study to date exploring the relationship between BMI and a large panel of markers for tumour phenotype in endometrial carcinoma. The large sample size with careful characterisation of FIGO stage, histological subtype, and grade confers more accuracy to the estimates for the independent prognostic impact of BMI compared with smaller previous studies. Also, the fact that the patient series studied was derived from a well-defined geographic region in Norway, previously shown to be representative for the total Norwegian population (Salvesen *et al*, 1999), suggests that the findings may be representative for a Caucasian patient population in general.

We found a positive association between high BMI and favourable DSS in univariate analysis but not in multivariate analysis. However, in multivariate analysis of OS, we found an independent unfavourable prognostic impact of increasing BMI. Previous studies exploring the effect of BMI on survival have reported conflicting results, which may be due to sample sizes, choice of cut point for BMI, outcome variables applied, and the panel of clinico-pathological markers adjusted for in the multivariate analyses. Like the present study, several have reported a trend towards better survival in the overweight compared with the more slender women (Anderson *et al*, 1996; Temkin *et al*, 2007; Munstedt *et al*, 2008). Others have concluded with no difference (Jeong *et al*, 2010) and even poorer survival for women with higher BMI (von Gruenigen *et al*, 2006). Disease-specific survival applied in the present study is more likely to be accurate in detecting deaths directly related to the disease studied. Previous studies, mostly applying OS, may have underestimated the positive biological impact of obesity, as obese women have increased risk of dying from intercurrent disease (Anderson *et al*, 1996; Temkin *et al*, 2007). Our findings that OS is less favourable for obese women when adjusted for the standard clinico-pathological risk factors may support this.

A limit of our study is that BMI is measured at the time of diagnosis. This may lead to a bias, as aggressive cancers often are associated with weight loss, cachexia, and anorexia (Keller 1993). Hence, we may have underestimated the weight of patients presenting with high stage cancers.

The rise in endometrial carcinoma incidence has been associated with an epidemic of obesity and physical inactivity (Amant *et al*, 2005). Unopposed oestrogen exposure leads to endometrial hyperplasia, and increased risk of atypical hyperplasia and type I endometrial cancer (Shang, 2006). The significance of progesterone in controlling oestrogen-driven proliferation is underlined by its efficacy in preventing endometrial cancer (Kim and Chapman-Davis, 2010). Still, the molecular basis and cross talk between hormone receptor pathways are poorly understood (Kim and Chapman-Davis, 2010). In previous smaller immunohistochemical studies (Duska *et al*, 2001; Gates *et al*, 2006), no significant relationship between hormone receptor status and BMI was identified (*n*=41 and *n*=165, respectively). We found that BMI was significantly linked to alterations in PR but not ER*α* status in tumours, confirmed by two different techniques estimating mRNA and protein levels for PR and ER*α*. The biological function of PR may be altered by genetic variations. Interestingly, recent studies have identified a single-nucleotide polymorphism in the gene coding for the PR, which has been associated with increased risk for endometrial carcinoma (Xu *et al*, 2009; O’Mara *et al*, 2010). This support the complexity in the hormone receptor interactions related to carcinogenesis and tumour development in endometrial cancer, and further studies of these interactions are needed.

## Figures and Tables

**Table 1:** Distribution of clinico-pathological factors in 949 patients with endometrial carcinoma according to body mass index (BMI)

**Variable**	**Total no. of patients**	**Median BMI**	**Lean (%)**	**Normal (%)**	**Overweight (%)**	**Obese (%)**	***P*-value^a^**
*Age, quartiles* b	949						0.002
1 (age 26–58)		25.6	5 (2)	109 (45)	66 (27)	65 (27)	
2 (age 58–66)		27.1	8 (3)	76 (32)	82 (34)	73 (31)	
3 (age 66–74)		27.3	2 (1)	76 (31)	91 (37)	76 (31)	
4 (age 74–95)		25.1	8 (4)	97 (44)	70 (32)	45 (21)	
							
*Menopause* c	949						0.116
Pre/peri		26.1	1 (1)	54 (44)	31 (25)	38 (31)	
Post		26.4	22 (3)	304 (37)	278 (34)	221 (27)	
							
*FIGO stage*	949						<0.0001
I		26.6	10 (2)	246 (36)	224 (33)	197 (29)	
II		27.3	3 (3)	30 (29)	45 (44)	26 (25)	
III		24.4	7 (6)	55 (49)	30 (27)	21 (19)	
IV		24.0	3 (6)	27 (49)	10 (18)	15 (27)	
							
*Histological subtype*	949						
Endometrioid		26.6	16 (2)	297 (37)	269 (33)	229 (28)	0.030
Non-endometrioid		25.1	7 (5)	61 (44)	40 (29)	30 (22)	
							
*Grade* d	905						0.174
1 or 2		26.7	14 (2)	242 (36)	224 (34)	188 (28)	
3		25.7	9 (4)	99 (42)	71 (30)	58 (25)	
							
*PR*	433						0.003^e^
Positive		26.9					
Negative		25.5					
							
*ER*	437						0.08^e^
Positive		26.7					
Negative		25.5					

Abbreviation: FIGO=International Federation of Gynaecology and Obstetrics.

a *χ*^2^-test when no other specified.

b Truncated to closest integer.

c Menopausal status was determined based on the information from the patient records.

d Data missing for 44 patients.

e Mann–Whitney *U*-test.

**Table 2:** Univariate survival analysis (Kaplan–Meier estimates) according to clinico-pathological factors and BMI in 1129 endometrial carcinoma patients

**Variable**	**No. of patients (no. of deaths)^a^**	**5-year survival**	***P* (log-rank)**
*Age, quartiles* b			<0.0001
1 (age 27–58)	282 (17)	94.5	
2 (age 58–66)	282 (44)	84.4	
3 (age 66–74)	283 (73)	73.8	
4 (age 74–94)	282 (89)	63.8	
Sum	1129		
			
*Menopausal status*			<0.0001
Pre/peri	145 (13)	93.9	
Post	983 (87)	77.1	
Sum^c^	1128		
			
*FIGO stage*			<0.0001
I	812 (79)	90.8	
II	119 (27)	74.2	
III	132 (68)	39.4	
IV	65 (48)	16.3	
Sum^d^	1128		
			
*Histological subtype*			<0.0001
Endometrioid	966 (146)	84.4	
Non-endometrioid	163 (77)	46.8	
Sum	1129		
			
*Grade*			<0.0001
1	345 (26)	92.0	
2	454 (81)	82.6	
3	283 (105)	56.9	
Sum^e^	1082		
			
*BMI WHO*			0.066^f^
Underweight (<18.5)	23 (7)	63.3	
Normal (18.5–24.9)	358 (77)	77.0	
Overweight (25–29.9)	309 (51)	81.9	
Obese (⩾30)	259 (47)	81.1	
Sum^g^	949		
			
*BMI quartiles*			0.096^f^
1 (14.7–23.1)	237 (54)	75.3	
2 (23.1–26.3)	240 (46)	79.1	
3 (26.3–30.5)	236 (39)	81.3	
4 (30.5–73.0)	236 (43)	81.4	
Sum	949		
			
*BMI 2 groups* h			0.035^f^
<25	381 (84)	76.3	
⩾25	568 (98)	81.6	
Sum	949		

Abbreviations: BMI=body mass index; FIGO=International Federation of Gynaecology and Obstetrics; WHO=World Health Organization.

a Number of patients varies due to missing data.

b Truncated to closest integer.

c Data for menopausal status missing for one patient.

d Data for FIGO stage missing for one patient.

e Data for grade missing for 67 patients.

f *P*-value with linear trend test.

g Data for BMI missing for 180 patients.

h Endometrioid carcinomas only: 5-year survival: BMI<25=81.2%, BMI⩾25=85.6% (*P*=0.134).

**Table 3:** Survival analysis of 905 endometrial carcinoma patients based on the Cox proportional hazards model

**Variable**	**No. of patients (%)**	**Unadjusted HR^a^**	**95% CI**	***P*-value**	**Adjusted HR**	**95% CI**	***P*-value**
*FIGO stage*				<0.0001			<0.0001
I	646 (71)	1.00			1.00		
II	96 (11)	3.25	1.98–5.31		2.83	1.72–4.65	
III	111 (12)	9.75	6.73–14.12		8.13	5.52–11.97	
IV	52 (6)	32.60	21.10–50.35		24.41	14.80–40.26	
							
*Histological subtype*				<0.0001			0.08
Endometrioid	777 (86)	1.00			1.00		
Non-endometrioid	128 (14)	4.76	3.44–6.57		1.49	0.95–2.32	
							
*Grade*				<0.0001			0.11
1 or 2	668 (74)	1.00			1.00		
3	237 (26)	3.84	2.83–5.19		1.41	0.93–2.13	
*Age* b	904 (100)	1.06	1.04–1.07	<0.0001	1.05	1.03–1.06	<0.0001
							
*BMI* c				0.04			0.65
<25	364 (40)	1.38	1.02–1.86		0.93	0.68–1.27	
⩾25	541 (60)	1.00			1.00		

Abbreviations: BMI=body mass index; CI=confidence interval; FIGO=International Federation of Gynaecology and Obstetrics; HR=hazard ratio.

a Analyses based on patients with complete information for all variables (*n*=905).

b Age at primary operation, continuous variable with HR given per year.

c When including patients with endometrioid histology only: adjusted HR for BMI was 1.07, 95% CI 0.73–1.55, *P*=0.7.
